# Supplementary material for: Assessing Chinese anatomists’ perceptions and attitudes toward blended learning through faculty development training programs
Source: PeerJ. 2023 Oct 30;11:e16283. doi: 10.7717/peerj.16283 (PMC10621592; doi:10.7717/peerj.16283)
Supplement: Apendix S1 [file peerj-11-16283-s002.docx]

Appendix 1. The English translated version of the questionnaire.

**The questionnaire for anatomy education and faculty development programs during COVID-19 pandemic**

We sincerely thank you for participating in this national survey on anatomy education, which is organized by the Chinese Society for Anatomical Sciences. The results will provide a summary of our current efforts in organizing anatomy education, identifying problems we are facing, and even helping to improve the design and quality of anatomy education in the future. The questionnaire contains 59 questions and will take about 8-10 minutes to complete. There are no standard answers for these questions. Please answer them based on your experience of anatomy teaching and faculty training programs ever during the COVID-19 pandemic. We pledge to keep all data anonymous, and personal information will not be disclosed. Thank you very much again for your participation.

*********************************************************************

**I. The respondents’ demographic information**

Your gender? [Single choice] *

| ○ Male |
| --- |
| ○ Female |

Your age? [Please fill in the space below] *

_________________________________

Which kind of university/college do you work at? [Single choice] *

| ○ Public school |
| --- |
| ○ Private school  ○ Other (explain please) |

What job title do you have at your university/college? [Single choice] *

| ○ Post-doctor |
| --- |
| ○ Assistant professor  ○ Lecture  ○ Associate professor  ○ Professor  How long have you worked at your current university/college? [Please fill in the space below] *  _________________________________  What academic degrees have you been rewarded? [Multiple choices] *  ○ PhD  ○ MD  ○ Master degree  ○ Bachelor degree  ○ Other (explain please)  When did you start your anatomy teaching? [Single choice] *  ○ Before 2005  ○ Between 2005 and 2010  ○ Between 2011 and 2015  ○ Between 2016 and 2021  *********************************************************************  **II. The experience as an anatomy educator**  Please choose the discipline and students’ major you teach. [Matrix with multiple choice] *   \|  \| Medicine \| Pharmacy \| Others relevant to healthcare \| Other majors \| \| --- \| --- \| --- \| --- \| --- \| \| Clinical anatomy \| ○ \| ○ \| ○ \| ○ \| \| Neuroanatomy \| ○ \| ○ \| ○ \| ○ \| \| Histology \| ○ \| ○ \| ○ \| ○ \| \| Cell biology \| ○ \| ○ \| ○ \| ○ \| \| Forensic medicine \| ○ \| ○ \| ○ \| ○ \| \| Comparative anatomy \| ○ \| ○ \| ○ \| ○ \| \| Veterinary anatomy \| ○ \| ○ \| ○ \| ○ \| \| Embryology/Developmental anatomy \| ○ \| ○ \| ○ \| ○ \| \| Others (explain please) \| ○ \| ○ \| ○ \| ○ \|   What is/are your research fields? [Multiple choices] *  ○ Morphological anatomy  ○ Histology and cell biology  ○ Embryology and developmental anatomy  ○ Clinical and functional anatomy  ○ Radiology  ○ Anthropobiology  ○ Anthropology  ○ Medical/anatomical education  ○ Others (explain please)  *********************************************************************  **III. The professional training received as a teacher and a researcher**  What kind of professional training related to education have you had since you worked as a teacher at university/college? [Single choice] *  ○ Formal training (e.g., teacher training courses/programs)  ○ Informal training (e.g., guided by senior teachers/colleagues)  ○ Not confirmed  Have your division/department arranged a mentor for you since you worked as a teacher? [Single choice] *  ○ Yes  ○ No  Do you think that you have been fully supported from your university/college since you became a university staff? (1: not at all; 6: fully supported) [Single choice] *  ○ 1 ○ 2 ○ 3 ○ 4 ○ 5 ○ 6  Which activity below is mostly contributed to support your career development? (1: completely useless; 6: very useful) [Matrix multi-point scale question] *   \|  \| 1 \| 2 \| 3 \| 4 \| 5 \| 6 \| \| --- \| --- \| --- \| --- \| --- \| --- \| --- \| \| Attending academic conference \| ○ \| ○ \| ○ \| ○ \| ○ \| ○ \| \| Guidance from adviser \| ○ \| ○ \| ○ \| ○ \| ○ \| ○ \| \| Learning from colleagues \| ○ \| ○ \| ○ \| ○ \| ○ \| ○ \| \| Discussing with colleagues \| ○ \| ○ \| ○ \| ○ \| ○ \| ○ \| \| Workshop/training courses etc. \| ○ \| ○ \| ○ \| ○ \| ○ \| ○ \| \| Visiting scholar \| ○ \| ○ \| ○ \| ○ \| ○ \| ○ \|   How much do you think that the following choices could contribute to your career development? (1: completely useless; 6: very useful) [Matrix multi-point scale question] *   \|  \| 1 \| 2 \| 3 \| 4 \| 5 \| 6 \| \| --- \| --- \| --- \| --- \| --- \| --- \| --- \| \| International collaboration with foreign anatomists on education \| ○ \| ○ \| ○ \| ○ \| ○ \| ○ \| \| International collaboration with foreign anatomists on scientific research \| ○ \| ○ \| ○ \| ○ \| ○ \| ○ \| \| Training of anatomical knowledge \| ○ \| ○ \| ○ \| ○ \| ○ \| ○ \| \| Obtaining anatomical knowledge via VR or online learning \| ○ \| ○ \| ○ \| ○ \| ○ \| ○ \| \| Training the skill to use scientific resources (equipment and information etc. ) \| ○ \| ○ \| ○ \| ○ \| ○ \| ○ \| \| Gaining the support to attend scientific conference \| ○ \| ○ \| ○ \| ○ \| ○ \| ○ \| \| Training how to organize conference or workshop \| ○ \| ○ \| ○ \| ○ \| ○ \| ○ \| \| Training how to write scientific manuscript \| ○ \| ○ \| ○ \| ○ \| ○ \| ○ \| \| Training how to apply for research grant \| ○ \| ○ \| ○ \| ○ \| ○ \| ○ \| \| Training how to make Poster \| ○ \| ○ \| ○ \| ○ \| ○ \| ○ \| \| Training how to prepare CV \| ○ \| ○ \| ○ \| ○ \| ○ \| ○ \| \| Training how to cultivate leadership \| ○ \| ○ \| ○ \| ○ \| ○ \| ○ \| \| Cultivating management ability (teamwork, financial, project management etc.) \| ○ \| ○ \| ○ \| ○ \| ○ \| ○ \| \| Training how to implement a research project \| ○ \| ○ \| ○ \| ○ \| ○ \| ○ \| \| Training how to implement popularization of science \| ○ \| ○ \| ○ \| ○ \| ○ \| ○ \| \| Training research ability (e.g., microscopy, cell culture and statistics etc.) \| ○ \| ○ \| ○ \| ○ \| ○ \| ○ \|   Do you have the opportunity to collaborate with the foreign anatomists on scientific project or education? (1: not at all; 6: fully supported) [Single choice] *  ○ 1 ○ 2 ○ 3 ○ 4 ○ 5 ○ 6  What is your opinion about the benefit from holding the academic membership (e.g., anatomical association) for your career development? (1: completely useless; 6: very useful) [Single choice] *  ○ 1 ○ 2 ○ 3 ○ 4 ○ 5 ○ 6  Do you have funding to support your international conference or other academic communication activities? [Single choice] *  ○ None  ○ Occasionally  ○ Often have  ○ Always have  Have you had training experience on online teaching before Covid-19 pandemic? [Matrix multi-point scale question] *   \|  \| None \| Occasionally have \| Often have \| \| --- \| --- \| --- \| --- \| \| About network technology \| ○ \| ○ \| ○ \| \| About educational theory \| ○ \| ○ \| ○ \| \| Others \| ○ \| ○ \| ○ \|   Have you had training experience on online teaching ever since the beginning of pandemic in 2020? [Matrix multi-point scale question] *   \|  \| None \| Occasionally have \| Often have \| \| --- \| --- \| --- \| --- \| \| About network technology \| ○ \| ○ \| ○ \| \| About educational theory \| ○ \| ○ \| ○ \| \| Others \| ○ \| ○ \| ○ \|   Do you think the training about how to implement online teaching helpful? (1: completely helpless; 6: very helpful) [Single choice] *  ○ 1 ○ 2 ○ 3 ○ 4 ○ 5 ○ 6  Have you had the experience of online assessments? [Matrix multi-point scale question] *   \|  \| Never \| Have \| \| --- \| --- \| --- \| \| Before the Covid-19 pandemic \| ○ \| ○ \| \| Ever since the beginning of pandemic in 2020 \| ○ \| ○ \|   Have you received the training on the online assessments? [Matrix multi-point scale question] *   \|  \| Never \| Occasionally have \| Often have \| \| --- \| --- \| --- \| --- \| \| Before the Covid-19 pandemic \| ○ \| ○ \| ○ \| \| Ever since the beginning of pandemic in 2020 \| ○ \| ○ \| ○ \|   Have you implemented online anatomy education? [Matrix multi-point scale question] *   \|  \| Never \| Occasionally have \| Often have \| \| --- \| --- \| --- \| --- \| \| Before the Covid-19 pandemic \| ○ \| ○ \| ○ \| \| Ever since the beginning of pandemic in 2020 \| ○ \| ○ \| ○ \|   Blended learning approach have already become a kind of normal teaching format ever since COVID-19. So far, how many percentage of online teaching in your blended learning course? [Single choice] *  ○ All face-to-face (F2F) class  ○ 1-30%  ○ 30-50%  ○ 50-80%  ○ >80% |

Concerning about blended learning, it includes online learning and F2F class, learners could study anytime, anywhere, as well as by any learning methods. Compared to traditional F2F class, please provide your evaluation about how well the blended learning works. [Single choice] *

○ Worse than traditional F2F class

○ Learning outcome is similar between them

○ Better than traditional F2F class

*********************************************************************

**IV. The readiness towards blended learning**

**Please answer the following questions related to "Blended Learning" based on your perceptions. (1: Strongly disagree; 6: Strongly agree)**

1. I would like unlimited access to lecture materials. [Single choice] *

| ○1 | ○2 | ○3 | ○4 | ○5 | ○6 |
| --- | --- | --- | --- | --- | --- |

2. I would like to decide where I want to study. [Single choice] *

| ○1 | ○2 | ○3 | ○4 | ○5 | ○6 |
| --- | --- | --- | --- | --- | --- |

3. I like to study at my own pace. [Single choice] *

| ○1 | ○2 | ○3 | ○4 | ○5 | ○6 |
| --- | --- | --- | --- | --- | --- |

4. I would like to decide when I want to study. [Single choice] *

| ○1 | ○2 | ○3 | ○4 | ○5 | ○6 |
| --- | --- | --- | --- | --- | --- |

5. I believe face-to-face learning is more effective than online learning. [Single choice] *

| ○1 | ○2 | ○3 | ○4 | ○5 | ○6 |
| --- | --- | --- | --- | --- | --- |

6. I am comfortable with self-directed learning. [Single choice] *

| ○1 | ○2 | ○3 | ○4 | ○5 | ○6 |
| --- | --- | --- | --- | --- | --- |

7. I do not resist having my lessons online. [Single choice] *

| ○1 | ○2 | ○3 | ○4 | ○5 | ○6 |
| --- | --- | --- | --- | --- | --- |

8. I like online learning as it provides richer instructional content. [Single choice] *

| ○1 | ○2 | ○3 | ○4 | ○5 | ○6 |
| --- | --- | --- | --- | --- | --- |

9. I would like lecture time in the classroom to be reduced. [Single choice] *

| ○1 | ○2 | ○3 | ○4 | ○5 | ○6 |
| --- | --- | --- | --- | --- | --- |

10. I would like to have my classes online rather than in the classroom. [Single choice] *

| ○1 | ○2 | ○3 | ○4 | ○5 | ○6 |
| --- | --- | --- | --- | --- | --- |

11. I get bored when studying online. [Single choice] *

| ○1 | ○2 | ○3 | ○4 | ○5 | ○6 |
| --- | --- | --- | --- | --- | --- |

12. I find it very difficult to study online. [Single choice] *

| ○1 | ○2 | ○3 | ○4 | ○5 | ○6 |
| --- | --- | --- | --- | --- | --- |

13. I am more likely to miss assignment due dates in an online learning environment. [Single choice] *

| ○1 | ○2 | ○3 | ○4 | ○5 | ○6 |
| --- | --- | --- | --- | --- | --- |

14. I organize my time better when studying online. [Single choice] *

| ○1 | ○2 | ○3 | ○4 | ○5 | ○6 |
| --- | --- | --- | --- | --- | --- |

15. I can study over and over again online. [Single choice] *

| ○1 | ○2 | ○3 | ○4 | ○5 | ○6 |
| --- | --- | --- | --- | --- | --- |

16. Online learning motivates me to prepare well for my studies. [Single choice] *

| ○1 | ○2 | ○3 | ○4 | ○5 | ○6 |
| --- | --- | --- | --- | --- | --- |

17. Online learning encourages me to make plans. [Single choice] *

| ○1 | ○2 | ○3 | ○4 | ○5 | ○6 |
| --- | --- | --- | --- | --- | --- |

18. Online learning makes me more responsible for my studies. [Single choice] *

| ○1 | ○2 | ○3 | ○4 | ○5 | ○6 |
| --- | --- | --- | --- | --- | --- |

19. I believe the Web is a useful platform for learning. [Single choice] *

| ○1 | ○2 | ○3 | ○4 | ○5 | ○6 |
| --- | --- | --- | --- | --- | --- |

20. I am familiar with Web technologies. [Single choice] *

| ○1 | ○2 | ○3 | ○4 | ○5 | ○6 |
| --- | --- | --- | --- | --- | --- |

21. I find Web technologies easy to use. [Single choice] *

| ○1 | ○2 | ○3 | ○4 | ○5 | ○6 |
| --- | --- | --- | --- | --- | --- |

22. I think we should use technologies in learning. [Single choice] *

| ○1 | ○2 | ○3 | ○4 | ○5 | ○6 |
| --- | --- | --- | --- | --- | --- |

23. I have a sense of community when I meet other students in the classroom. [Single choice] *

| ○1 | ○2 | ○3 | ○4 | ○5 | ○6 |
| --- | --- | --- | --- | --- | --- |

24. I like the fast feedback when I meet my lecturer in person. [Single choice] *

| ○1 | ○2 | ○3 | ○4 | ○5 | ○6 |
| --- | --- | --- | --- | --- | --- |

25. I find learning through collaboration with others face-to-face is more effective. [Single choice] *

| ○1 | ○2 | ○3 | ○4 | ○5 | ○6 |
| --- | --- | --- | --- | --- | --- |

1. I learn better through lecturer-directed classroom-based activities. [Single choice] *

| ○1 | ○2 | ○3 | ○4 | ○5 | ○6 |
| --- | --- | --- | --- | --- | --- |

1. I learn better when someone guides me personally. [Single choice] *

| ○1 | ○2 | ○3 | ○4 | ○5 | ○6 |
| --- | --- | --- | --- | --- | --- |

1. I feel isolated in an online learning environment. [Single choice] *

| ○1 | ○2 | ○3 | ○4 | ○5 | ○6 |
| --- | --- | --- | --- | --- | --- |

1. I am comfortable in using Web technologies to exchange knowledge with others. [Single choice] *

| ○1 | ○2 | ○3 | ○4 | ○5 | ○6 |
| --- | --- | --- | --- | --- | --- |

1. I would like to interact with my lecturer online. [Single choice] *

| ○1 | ○2 | ○3 | ○4 | ○5 | ○6 |
| --- | --- | --- | --- | --- | --- |

1. I would like to interact with other students outside of the classroom. [Single choice] *

| ○1 | ○2 | ○3 | ○4 | ○5 | ○6 |
| --- | --- | --- | --- | --- | --- |

1. I find it easy to communicate with others online. [Single choice] *

| ○1 | ○2 | ○3 | ○4 | ○5 | ○6 |
| --- | --- | --- | --- | --- | --- |

1. I appreciate easy online access to my lecturer. [Single choice] *

| ○1 | ○2 | ○3 | ○4 | ○5 | ○6 |
| --- | --- | --- | --- | --- | --- |

1. I can collaborate well with a virtual team in doing assignments. [Single choice] *

| ○1 | ○2 | ○3 | ○4 | ○5 | ○6 |
| --- | --- | --- | --- | --- | --- |
