# Supplementary material for: Assessing Chinese anatomists’ perceptions and attitudes toward blended learning through faculty development training programs
Source: PeerJ. 2023 Oct 30;11:e16283. doi: 10.7717/peerj.16283 (PMC10621592; doi:10.7717/peerj.16283)
Supplement: Appendix S2 [file peerj-11-16283-s003.docx]

**国际解剖学工作者协会联盟（IFAA）关于线上教学的问卷[复制]**

各位同仁，大家好！

时值中国解剖学会成立百年之际，国际解剖学工作者协会联盟（IFAA）也拟在世界解剖学日（World Anatomy Day）期间开展包括下列问卷调查在内的系列活动。

本问卷为IFAA针对COVID-19疫情之后，就解剖学老师开展的线上教学、混合式教学、得到的教学培训等情况进行的一项调查。原卷为英文，为了减轻大家的负担、便于理解、节省时间和方便回答，我们将其翻译成了中文。调查结束之后，我们将汇总大家的答案，向IFAA报告。本问卷为匿名问卷，不会泄露任何个人信息，请您按照自己的情况如实填写。

本问卷可以直接在线填写。也可以填写了word表格发回到邮箱：3579310@qq.com。
如有问题，请您拨打电话：13802978198

谢谢您的支持、配合和帮助！

中国解剖学会国际交流委员会
2021年10月14日

1. 您的性别 [单选题] *

| ○男性 |
| --- |
| ○女性 |

2. 您的年龄 [填空题] *

_________________________________

3. 您工作的院校是 [单选题] *

| ○公立院校 |
| --- |
| ○民办院校 |
| ○其它（请说明） _________________ |

4. 您的工作身份是 [单选题] *

| ○博士后 |
| --- |
| ○助教 |
| ○讲师 |
| ○副教授 |
| ○教授 |
| ○其它（请说明） _________________ * |

5. 您在现单位工作多少年了？ [填空题] *

_________________________________

6. 您的学历是 [多选题] *

| □PhD |
| --- |
| □MD |
| □硕士学位 |
| □学士学位 |
| □其它（请说明） _________________* |

7. 您什么时候开始解剖学教学的 [单选题] *

| ○2005年之前 |
| --- |
| ○2005-2010 |
| ○2011-2015 |
| ○2016-2021 |

8. 请您选择您教学的领域及所教学生的专业：[矩阵单选题]

|  | 医学专业 | 药学专业 | 其它与健康相关的专业 | 其它学生 |
| --- | --- | --- | --- | --- |
| 临床解剖学 | ○ | ○ | ○ | ○ |
| 神经解剖学 | ○ | ○ | ○ | ○ |
| 组织学 | ○ | ○ | ○ | ○ |
| 细胞生物学 | ○ | ○ | ○ | ○ |
| 法医学 | ○ | ○ | ○ | ○ |
| 比较解剖学 | ○ | ○ | ○ | ○ |
| 兽医解剖学 | ○ | ○ | ○ | ○ |
| 胚胎学/发育解剖学 | ○ | ○ | ○ | ○ |
| 其它（请说明） | ○ | ○ | ○ | ○ |

9. 请选择您的研究领域 [多选题] *

| □形态解剖学 |
| --- |
| □组织学，细胞生物学 |
| □胚胎学和发育解剖学 |
| □临床解剖学或功能解剖学 |
| □口腔生物学 |
| □影像学 |
| □人类生物学 |
| □人类学 |
| □医学/解剖学教育 |
| □其他（请说明） _________________ |

10. 作为一名教师，您接受过的教育相关的职业培训是： [单选题] *

| ○正式培训（如参加过教师培训课程/培训项目等） |
| --- |
| ○非正式培训（如其他高年资教师或同事指导） |
| ○不确认 |

11. 您从事教学工作之后，科室或学系给您配备了指导老师么？ [单选题] *

| ○有 |
| --- |
| ○没有 |

12. 从成长为一个本专业的优秀教育工作者来说，您觉得是否得到了足够的支持？
（1: 完全没有；6: 得到充分支持） [单选题] *

| ○1 | ○2 | ○3 | ○4 | ○5 | ○6 |
| --- | --- | --- | --- | --- | --- |

13. 您觉得下面哪一项活动对您职业发展效果最明显？
（1: 完全没有效果；6: 非常有效果）[矩阵量表题] *

|  | 1 | 2 | 3 | 4 | 5 | 6 |
| --- | --- | --- | --- | --- | --- | --- |
| 参加学术会议 | ○ | ○ | ○ | ○ | ○ | ○ |
| 有导师指导 | ○ | ○ | ○ | ○ | ○ | ○ |
| 观察同事、同行 | ○ | ○ | ○ | ○ | ○ | ○ |
| 与同事、同行讨论 | ○ | ○ | ○ | ○ | ○ | ○ |
| 工作坊、培训课程等 | ○ | ○ | ○ | ○ | ○ | ○ |
| 访问学者 | ○ | ○ | ○ | ○ | ○ | ○ |

14. 下面选项中，您认为获得更多信息能对职业发展更有利的程度分别是？
（1: 完全没有用；6: 非常有利）[矩阵量表题] *

|  | 1 | 2 | 3 | 4 | 5 | 6 |
| --- | --- | --- | --- | --- | --- | --- |
| 与国际解剖学者在教学方面的联系与合作 | ○ | ○ | ○ | ○ | ○ | ○ |
| 与国际解剖学者在科研方面的联系与合作 | ○ | ○ | ○ | ○ | ○ | ○ |
| 解剖专业知识的培训 | ○ | ○ | ○ | ○ | ○ | ○ |
| 通过虚拟或者在线学习的方式获取解剖专业知识的培训 | ○ | ○ | ○ | ○ | ○ | ○ |
| 用于培训或者科研的一些设备、信息、资源等 | ○ | ○ | ○ | ○ | ○ | ○ |
| 获得参加会议的资助 | ○ | ○ | ○ | ○ | ○ | ○ |
| 关于如何组织会议或者工作坊的培训 | ○ | ○ | ○ | ○ | ○ | ○ |
| 科研论文写作培训 | ○ | ○ | ○ | ○ | ○ | ○ |
| 关于基金申请和培训课程的信息 | ○ | ○ | ○ | ○ | ○ | ○ |
| Poster制作 | ○ | ○ | ○ | ○ | ○ | ○ |
| 准备个人简历 | ○ | ○ | ○ | ○ | ○ | ○ |
| 培养领导力 | ○ | ○ | ○ | ○ | ○ | ○ |
| 培养管理技能（团队管理、经费管理、项目管理等） | ○ | ○ | ○ | ○ | ○ | ○ |
| 组织和实施科研项目 | ○ | ○ | ○ | ○ | ○ | ○ |
| 科普写作培训 | ○ | ○ | ○ | ○ | ○ | ○ |
| 科研技能（如显微照相、细胞培养、统计等） | ○ | ○ | ○ | ○ | ○ | ○ |

15. 您是否有与其他国家的解剖学工作者建立联系和合作的机会？
（1: 完全没有；6: 非常充分） [单选题] *

| ○1 | ○2 | ○3 | ○4 | ○5 | ○6 |
| --- | --- | --- | --- | --- | --- |

16. 您认为成为某个组织的成员（如解剖学会）或者有一个平台对于全球的解剖学工作者交流和合作的帮助程度是：
（1: 完全没有帮助；6：非常有帮助） [单选题] *

| ○1 | ○2 | ○3 | ○4 | ○5 | ○6 |
| --- | --- | --- | --- | --- | --- |

17. 您是否有经费参加会议或者其他交流活动？ [单选题] *

| ○无 |
| --- |
| ○偶尔有 |
| ○经常有 |
| ○一直都有 |

18. 在COVID-19之前，您是否接受过在线教学的培训活动？[矩阵量表题] *

|  | 没有 | 偶尔有 | 经常有 |
| --- | --- | --- | --- |
| 网络技术方面 | ○ | ○ | ○ |
| 教育理论方面 | ○ | ○ | ○ |
| 其他 | ○ | ○ | ○ |

19. 在2020年之后，您是否接受过在线教学的培训活动？[矩阵量表题] *

|  | 没有 | 偶尔有 | 经常有 |
| --- | --- | --- | --- |
| 网络技术方面 | ○ | ○ | ○ |
| 教育理论方面 | ○ | ○ | ○ |
| 其他 | ○ | ○ | ○ |

20. 您认为针对在线教学的培训是否有效？
（1: 完全没有用；6: 非常有帮助） [单选题] *

| ○1 | ○2 | ○3 | ○4 | ○5 | ○6 |
| --- | --- | --- | --- | --- | --- |

21. 您是否开展过在线教学的评价活动？[矩阵量表题] *

|  | 没有 | 有 |
| --- | --- | --- |
| 在COVID-19之前 | ○ | ○ |
| 2020年之后 | ○ | ○ |

22. 您是否接受过在线学习评价方面的培训？[矩阵量表题] *

|  | 没有 | 偶尔有 | 经常有 |
| --- | --- | --- | --- |
| 在COVID-19之前 | ○ | ○ | ○ |
| 在2020年之后 | ○ | ○ | ○ |

23. 您是否开展过您所在专业的在线教学[矩阵量表题] *

|  | 没有 | 偶尔有 | 经常有 |
| --- | --- | --- | --- |
| 在COVID-19之前 | ○ | ○ | ○ |
| 2020年以后 | ○ | ○ | ○ |

24. 疫情后，混合式教学已经成为很多单位常态化的教学方式。

当前，您开展线上+线下混合式教学时，线上学时占全部学时比例为：： [单选题] *

| ○全部线下 |
| --- |
| ○1%-30% |
| ○30%-50% |
| ○51%-80% |
| ○80%以上 |

25. 混合式学习的概念，不仅包括线上学习+线下学习的结合，还应包括学习者能够采用不同时间、学习地点、学习路径和学习步调的学习方式。

与传统的课堂教学相比，您觉得自己开展的混合式教学的效果如何？ [单选题] *

| ○不及单纯的课堂教学 |
| --- |
| ○两者效果接近 |
| ○优于单纯的课堂教学 |

请您从一个**接受培训的学习者**的角度，回答以下与“混合式学习”有关的问题。
（1: 非常不同意；6: 非常同意）

26. 我希望能没有限制地、免费得到培训材料。 [单选题] *

| ○1 | ○2 | ○3 | ○4 | ○5 | ○6 |
| --- | --- | --- | --- | --- | --- |

27. 我希望能决定我想在哪里学习。 [单选题] *

| ○1 | ○2 | ○3 | ○4 | ○5 | ○6 |
| --- | --- | --- | --- | --- | --- |

28. 我希望按我自己的节奏学习。 [单选题] *

| ○1 | ○2 | ○3 | ○4 | ○5 | ○6 |
| --- | --- | --- | --- | --- | --- |

29. 我想决定自己什么时候要学习。 [单选题] *

| ○1 | ○2 | ○3 | ○4 | ○5 | ○6 |
| --- | --- | --- | --- | --- | --- |

30. 我相信面对面的学习比在线学习更有效。 [单选题] *

| ○1 | ○2 | ○3 | ○4 | ○5 | ○6 |
| --- | --- | --- | --- | --- | --- |

31. 我很适应自主学习。 [单选题] *

| ○1 | ○2 | ○3 | ○4 | ○5 | ○6 |
| --- | --- | --- | --- | --- | --- |

32. 我并不抗拒在网上上课。 [单选题] *

| ○1 | ○2 | ○3 | ○4 | ○5 | ○6 |
| --- | --- | --- | --- | --- | --- |

33. 我喜欢在线学习，因为它提供了更丰富的教学内容。 [单选题] *

| ○1 | ○2 | ○3 | ○4 | ○5 | ○6 |
| --- | --- | --- | --- | --- | --- |

34. 我希望减少课堂教学的时间。 [单选题] *

| ○1 | ○2 | ○3 | ○4 | ○5 | ○6 |
| --- | --- | --- | --- | --- | --- |

35. 我想在网上上课，而不是在实体课堂上课。 [单选题] *

| ○1 | ○2 | ○3 | ○4 | ○5 | ○6 |
| --- | --- | --- | --- | --- | --- |

36. 我在网上学习时会觉得很无聊。 [单选题] *

| ○1 | ○2 | ○3 | ○4 | ○5 | ○6 |
| --- | --- | --- | --- | --- | --- |

37. 我发现在线学习对我很困难。 [单选题] *

| ○1 | ○2 | ○3 | ○4 | ○5 | ○6 |
| --- | --- | --- | --- | --- | --- |

38. 在在线学习环境中，我更有可能错过作业截止日期。 [单选题] *

| ○1 | ○2 | ○3 | ○4 | ○5 | ○6 |
| --- | --- | --- | --- | --- | --- |

39. 我在网上学习的时候能更好地组织好时间。 [单选题] *

| ○1 | ○2 | ○3 | ○4 | ○5 | ○6 |
| --- | --- | --- | --- | --- | --- |

40. 我可以在网上反复学习同一内容。 [单选题] *

| ○1 | ○2 | ○3 | ○4 | ○5 | ○6 |
| --- | --- | --- | --- | --- | --- |

41. 在线学习激励我为学习做好准备。 [单选题] *

| ○1 | ○2 | ○3 | ○4 | ○5 | ○6 |
| --- | --- | --- | --- | --- | --- |

42. 在线学习激励我制定学习计划。 [单选题] *

| ○1 | ○2 | ○3 | ○4 | ○5 | ○6 |
| --- | --- | --- | --- | --- | --- |

43. 在线学习让我对我的学习更负责任。 [单选题] *

| ○1 | ○2 | ○3 | ○4 | ○5 | ○6 |
| --- | --- | --- | --- | --- | --- |

44. 我认为网络是一个有用的学习平台。 [单选题] *

| ○1 | ○2 | ○3 | ○4 | ○5 | ○6 |
| --- | --- | --- | --- | --- | --- |

45. 我很熟悉网络技术。 [单选题] *

| ○1 | ○2 | ○3 | ○4 | ○5 | ○6 |
| --- | --- | --- | --- | --- | --- |

46. 我发现网络技术很容易使用。 [单选题] *

| ○1 | ○2 | ○3 | ○4 | ○5 | ○6 |
| --- | --- | --- | --- | --- | --- |

47. 我认为我们应该在学习中使用技术。 [单选题] *

| ○1 | ○2 | ○3 | ○4 | ○5 | ○6 |
| --- | --- | --- | --- | --- | --- |

48. 当我在教室里遇到其他同学时，我就有一种团体的意识。 [单选题] *

| ○1 | ○2 | ○3 | ○4 | ○5 | ○6 |
| --- | --- | --- | --- | --- | --- |

49. 我喜欢见到老师本人，得到来自老师的快速反馈。 [单选题] *

| ○1 | ○2 | ○3 | ○4 | ○5 | ○6 |
| --- | --- | --- | --- | --- | --- |

50. 我发现通过与他人面对面的合作来学习更有效。 [单选题] *

| ○1 | ○2 | ○3 | ○4 | ○5 | ○6 |
| --- | --- | --- | --- | --- | --- |

51. 我通过老师指导的课堂活动会获得更好的学习效果。 [单选题] *

| ○1 | ○2 | ○3 | ○4 | ○5 | ○6 |
| --- | --- | --- | --- | --- | --- |

52. 当有人亲自指导我时，我会学得更好。 [单选题] *

| ○1 | ○2 | ○3 | ○4 | ○5 | ○6 |
| --- | --- | --- | --- | --- | --- |

53. 我在在线学习的环境中感到孤立。 [单选题] *

| ○1 | ○2 | ○3 | ○4 | ○5 | ○6 |
| --- | --- | --- | --- | --- | --- |

54. 我很乐意使用网络技术来与他人交换知识。 [单选题] *

| ○1 | ○2 | ○3 | ○4 | ○5 | ○6 |
| --- | --- | --- | --- | --- | --- |

55. 我愿意和老师在线交流。 [单选题] *

| ○1 | ○2 | ○3 | ○4 | ○5 | ○6 |
| --- | --- | --- | --- | --- | --- |

56. 我想和其他学生在课堂外互动。 [单选题] *

| ○1 | ○2 | ○3 | ○4 | ○5 | ○6 |
| --- | --- | --- | --- | --- | --- |

57. 我发现在网上和别人交流很容易。 [单选题] *

| ○1 | ○2 | ○3 | ○4 | ○5 | ○6 |
| --- | --- | --- | --- | --- | --- |

58. 我很喜欢能在网上容易地找到我的老师。 [单选题] *

| ○1 | ○2 | ○3 | ○4 | ○5 | ○6 |
| --- | --- | --- | --- | --- | --- |

59. 我可以很好地与一个虚拟团队合作来完成作业。 [单选题] *

| ○1 | ○2 | ○3 | ○4 | ○5 | ○6 |
| --- | --- | --- | --- | --- | --- |
